# Health care provision to migrants: what did we learn from the Ukraine, Afghan, and Syrian crises?

**DOI:** 10.1093/eurpub/ckac129.117

**Published:** 2022-10-25

**Authors:** A Veizis

**Affiliations:** Intersos, Lancet Migration European Hub, Athens, Greece

## Abstract

Dr Veizis was involved in the immediate health care provision to Ukrainian people fleeing to neighbouring countries including Poland and Moldova, as well as health care provision to Syrian people residing in Greece, working for the non-governmental organisation INTERSOS and previously MSF. He will discuss his experiences to date in terms of immediate and long-term provision to these groups and the role of both NGOs and mainstream health services in meeting the needs of forcibly displaced migrants to Europe.

